# Coronary arteritis as a cause of sudden cardiac death in a young girl

**DOI:** 10.4322/acr.2021.310

**Published:** 2021-08-20

**Authors:** Purnima Paliwal, Swasti Jain, Arvind Ahuja, Sachin Mittal, Devender Singh Chauhan

**Affiliations:** 1 Atal Bihari Vajpayee Institute of Medical Sciences, Dr. Ram Manohar Lohia Hospital, Department of Pathology, New Delhi, India; 2 Atal Bihari Vajpayee Institute of Medical Sciences, Dr. Ram Manohar Lohia Hospital, Department of Forensic Medicine, New Delhi, India

**Keywords:** Tuberculosis, Coronary vessels, Death, Sudden, Cardiac, Autopsy

## Abstract

A case of probable coronary arteritis in a young girl who died suddenly and unexpectedly is presented. The histologic presentation of the disorder is discussed, especially the differential diagnosis of arteritis of the coronary arteries with an emphasis on tuberculosis (TB). TB myocarditis with or without concomitant lung involvement is rare, and tubercular coronary arteritis without underlying pulmonary Koch’s disease is all the rarer. We herein describe a case where the cause of death was ascertained on post-mortem examination.

## INTRODUCTION

Tuberculosis (TB) is a leading infectious disease killer worldwide and one of the top 10 causes of death overall.^1^ Immunosuppressive conditions like HIV/AIDS have led to its raised prevalence and serious consequences. It has a predilection to affect the pulmonary system in 70-80% of the cases.^2^ The extrapulmonary sites that are commonly involved include lymph nodes, bones, and joints, brain, gastrointestinal and genitourinary systems.^3^ The cause of death, primarily being a respiratory failure in TB patients, is substantiated by the most common location being the lung, evidenced by extensive pulmonary changes in antemortem as well as post-mortem examination.^4^ However, in a case of cardiac TB without pulmonary involvement, the cause of death is usually established on autopsy findings.^5^ We describe a rare case of coronary arteritis, which was the cause of sudden death in a young girl.

## CASE REPORT

A 17-year-old female with no significant past medical history had a sudden episode of unconsciousness while at school. There was no history of indulging in heavy exercise or sudden emotional triggers. There was no history of fever, cough, reduced appetite, or weight loss. She was taken to a nearby hospital, where she was declared brought dead after failed resuscitation. The patient was referred to our institute for an autopsy to know the cause of sudden death at a young age.

## AUTOPSY PRESENTATION

**Figure 1:**
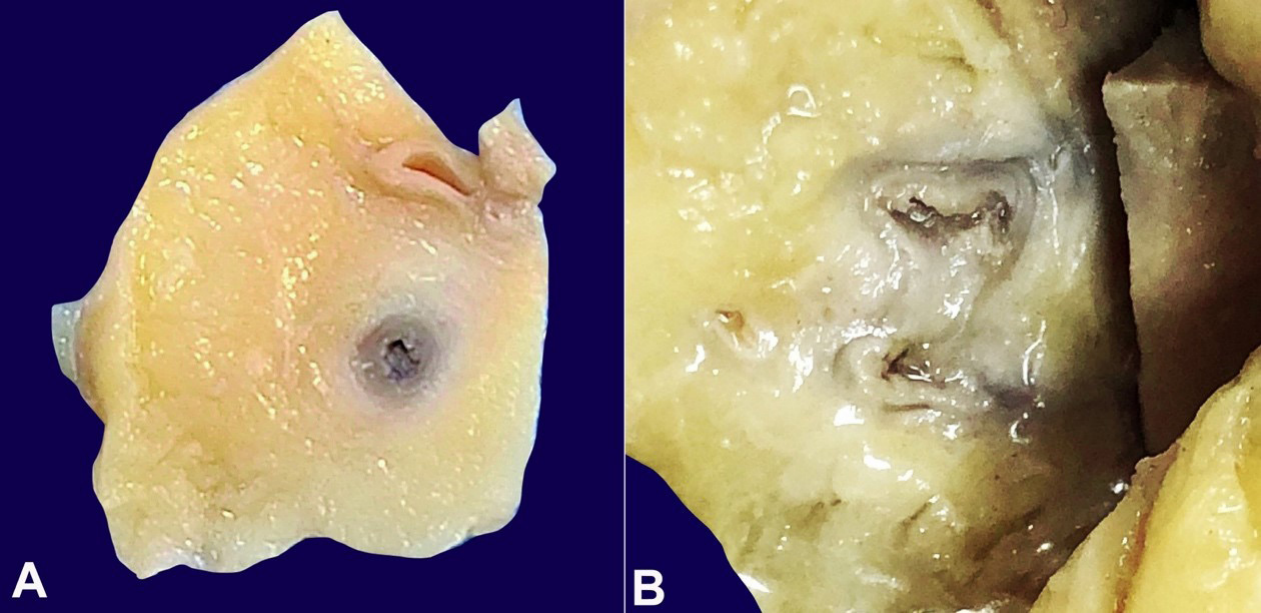
Gross view of the coronary arteries’ cut section. **A** – right coronary artery showing thickened wall and luminal narrowing; **B** – Gross image of left anterior descending coronary artery showing thickened gelatinous wall and partial obliteration of the lumen.

The body was that of a thin-built young female. No external signs of injury or swelling or any lymphadenopathy were identified. On the body’s opening, both the pleural cavities, peritoneal and pericardial sac, were free from any effusion, adhesions, or plaques. The heart was normal-sized, weighing 260 g (reference range [RR]; 148-296 g).^6^ Epicardial surface was shiny with no area of fibrosis. The coronary arteries were traceable and showed normal contouring. The left circumflex artery showed a fully patent lumen; however, the left anterior descending artery (LAD) and the right coronary artery showed a thickened gelatinous wall with >75% luminal occlusion in the LAD (Figure 1A, B).

The cut section of the heart did not reveal any infarct or discoloration. No stenosis, vegetations, or calcifications could be identified in the valves. There was no evidence of any septal defect. The left ventricular wall thickness measured 1.5 cm (RR; 1.2-1.5 cm), and the right ventricular wall measured 0.5cm (RR; 0.3-0.5 cm) in thickness.^6^ Rest of the major vessels, including the aorta, pulmonary trunk, and their main branches, were essentially unremarkable.

A large paratracheal lymph node was identified, measuring 1.8 cm in diameter, and showed a necrotic cut surface. Macroscopically, the remaining organs, including lungs, liver, spleen, kidney, pancreas, appeared unremarkable.

**Figure 2:**
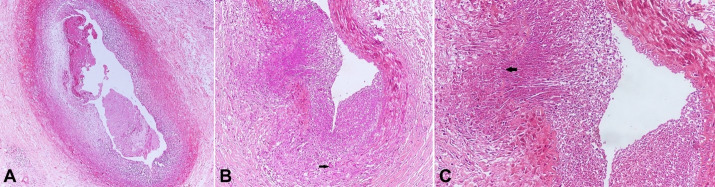
Photomicrographs of the coronary artery. **A** – right coronary showing non-occlusive fibrin thrombus and fibrointimal thickening with dense lymphohistiocytic infiltrate (H&E x 200); **B** – LAD showing luminal narrowing and dense transmural lymphohistiocytic infiltrate with occasional Langhans giant cell (arrow) (H&E x 100); **C** – Higher magnification of inflamed LAD to highlight focal necrosis and destruction of the elastic lamina (arrow) (H&E x 200).

Microscopic sections from the right coronary artery revealed a non-occlusive loose fibrin thrombus in the lumen. The arterial wall showed fibrointimal thickening with a dense chronic inflammatory infiltrate comprising of lymphocytes and histiocytes (Figure 2A). Sections from the left circumflex artery showed a patent lumen. Sections from the left anterior descending artery showed dense transmural lymphoplasmacytic inflammation in the vessel wall with the presence of patchy fibrosis. Focally, inflammation was seen extending into the surrounding adipose tissue. Focal necrosis and ill-defined histiocytic collection were also seen with occasional Langhans giant cells, however, the necrosis was found in all the serial sections (Figure 2B). There was focal fragmentation of the internal elastic lamina by the infiltrate (Figure 2C).

**Figure 3:**
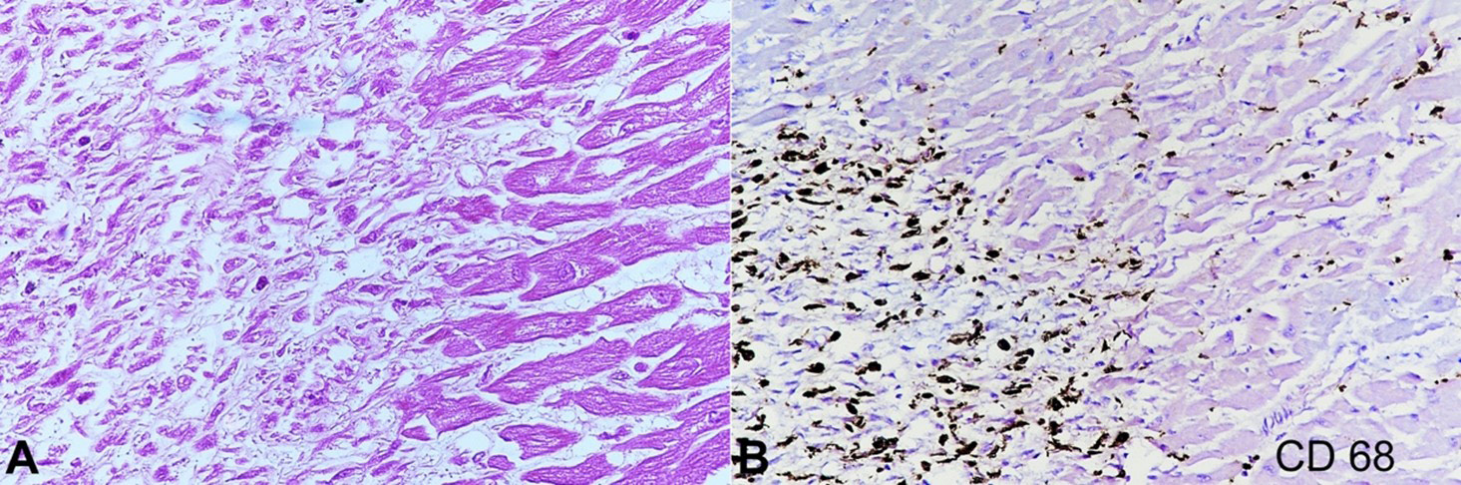
Photomicrograph of the myocardium. **A** – Patchy infiltrate of histiocytes within myocardial fibers (H&E, x 400); **B** – CD 68 positive infiltrate of histiocytes within myocardial fibers (X 200).

Multiple sections from grossly unremarkable myocardium revealed an occasional focus of histiocytic collection with some epithelioid cells (Figure 3A). These were immunoreactive to CD 68 (Figure 3B).

**Figure 4:**
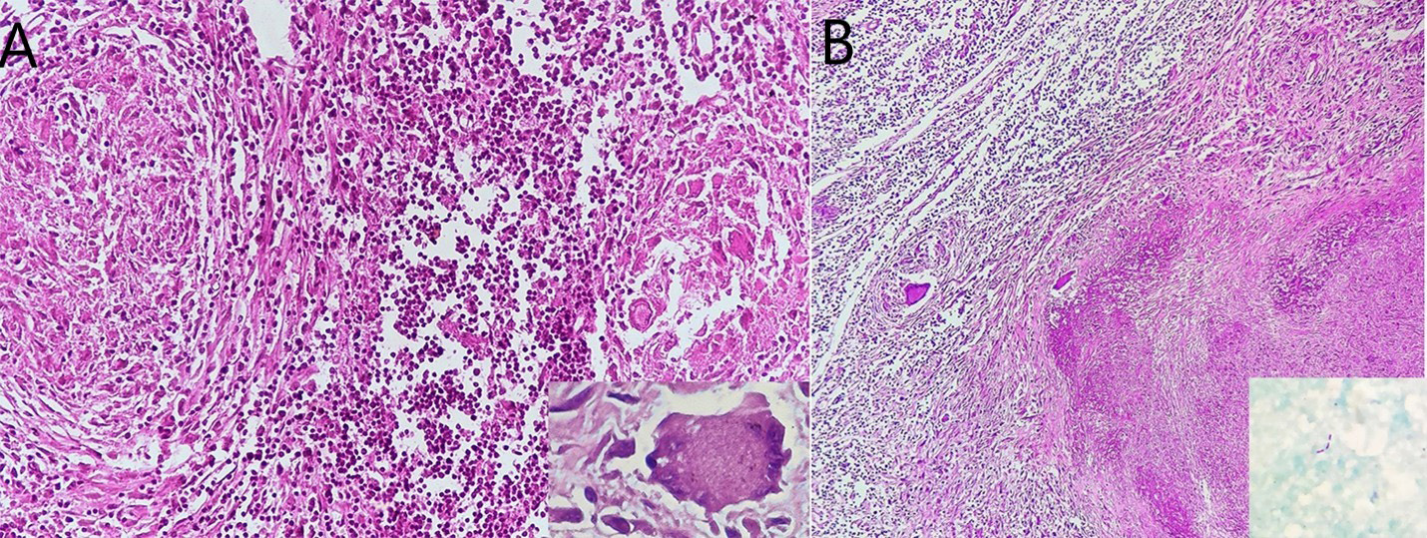
Photomicrograph of the lymph node. **A** – Epithelioid cell granulomas in the lymph node (Inset- multinucleated giant cell) (H&E x 200); **B** – Extensive caseous necrosis in paratracheal lymph node (H&E x 100). Acid-fast bacilli (Inset ZN x 1000).

Lymph node revealed extensive caseous necrosis rimmed by epithelioid cell granulomas, with interspersed Langhans giant cells (Inset) (Figure 4A, B). Few acid-fast bacilli were demonstrated on Ziehl-Neelsen (ZN) stain (Inset).

Sections from the brain, spinal cord, and brain stem appeared unremarkable. Kidney revealed the presence of an occasional epithelioid cell granuloma with Langhans giant cells in the interstitium. However, the Ziehl-Neelsen stain was non-contributory. Multiple sections from both lungs showed interstitial hemorrhage and mild emphysematous changes. No granulomas or giant cells could be demonstrated. Histology of the liver, spleen, and pancreas did not reveal any significant pathology.

## DISCUSSION

India is the highest TB burden country globally, having an estimated incidence of 26.9 x 10^5^ cases in 2019.^1^ Though TB can affect any organ of the human body, the most common presentation is usually of Pulmonary Koch’s disease. Extrapulmonary TB is most likely seen as lymphadenitis with other less common sites being the brain, gastrointestinal tract, genitourinary tract, bones and joints.^3^ The heart is involved only in 0.14-2% of all the TB cases, and it tends to affect the pericardium or the myocardium primarily.^7^ TB myocarditis was first reported in 1664 by Maurocordat.^8^ Cardiac TB can be morphologically categorized into three types: diffuse infiltrative, miliary, and tuberculomas.^7^ Rose et al.^5^ did an extensive study on 19 cases of cardiac TB, and every case demonstrated either miliary or systemic TB. The authors were convinced with three possible routes of spread to the myocardium: directly from the pleural cavity or the pericardium, spread from lymph nodes, and hematogenous dissemination.

The extension of the tuberculous disease into the myocardial vessels often occurs through the hematogenous spread from the lungs.^9^ However, tuberculous mediastinal lymphadenitis is also believed to be the cause of direct spread into the myocardium, from where it can involve the coronary arteries.^7^ Generally, the inflammation in the surrounding tissue tends to extend into the adventitial layer, morphologically seen as edema, mononuclear infiltrate and/or granulation tissue, and this further can spread till the intima.^10^ Fong et al.^11^ found that the mycobacteria directly invade the blood vessels of the heart leading to an autoimmune reaction and subsequently thrombosis. In the present case, the likely route of cardiac spread is lymphatic as the mediastinal lymph node revealed features of necrotizing granulomatous lymphadenitis. We cannot rule out the possibility of hematogenous seeding as the renal interstitium also showed the presence of granulomas. Another differential diagnosis that could be considered in this young girl is Takayasu arteritis, which primarily affects large vessels, such as the aorta and its main branches.^12^ It may also involve coronary arteries, resulting in gradual stenosis. Our case did not reveal any abnormality in the major vessels making the possibility of this disease unlikely.

The possible mechanisms of sudden cardiac death (SCD) in patients of TB myocarditis have been described in the literature as prolonged QT interval syndrome, ventricular tachycardia, heart failure, dilated cardiomyopathy, and right ventricular obstruction.^4^ Sudden death results mostly due to the arrhythmogenic process rather than the obstructive pathology. Liu^4^ suggested a possibility of minor coronary inflammation or spasms as the cause of ischemic heart disease in patients with TB-related sudden cardiac death. The first case of coronary arteritis was reported by Jacobs and Eliot in 1955.^13^ A similar case was then reported by Kinare et al.^10^ where a 19-year-old-boy had active tuberculous inflammation of the epicardium and the endocardium, which extended to the anterior descending coronary artery resulting in its occlusion. This was followed by a ventricular aneurysm due to myocardial infarction, which bulged into the outflow tract of the right ventricle producing pulmonary stenosis, and the patient died of a sudden attack of breathlessness.^10^ Another case of SCD related to tuberculous coronary arteritis was reported by Rodiguez et al.^14^ where heavy exercise was believed to have resulted in myocardial ischemia suggesting the variable reduction in the caliber of the coronary vessels due to TB inflammation. This was similar to the case by Chan et al.^7^ as both these patients had concomitant pulmonary TB. Table 1 summarizes the reported cases of sudden death due to tuberculous coronary arteritis. Predominantly young males were affected, associated pulmonary tuberculosis was seen in all but one case, and granulomatous inflammation of the left anterior descending artery was the most frequent. Ours is the only female patient without concomitant tuberculous pathology in the lungs.

**Table 1:** Review of cases of Tuberculous coronary arteritis on autopsy

	Age/Sex	Coronary inflammation	Artery involvement	Heart involvement	Synchronous involvement
Kinare^10^	19/M	Non- granulomatous	LAD	Epicardial	Mediastinal LN
Zlateva^9^	NA	Granulomatous inflammation	LCA	None	Lung
Rodriguez^14^	21/M	Granulomatous inflammation	NA	None	Lung
Chan S^7^	56/M	Granulomatous inflammation	RCA	Myocardial	Lung
Peddle^15^	37/M	Granulomatous inflammation	LCA	None	None
Index case	17/F	Granulomatous inflammation	LAD and RCA	Myocardial	Mediastinal LN

Age in years; F= female; LAD=Left anterior descending; LCA= left coronary artery; LN=Lymph node; M=male; NA= Not available; RC=right coronary artery.

In our patient, the cause of sudden death is probably the result of arrhythmia triggered by spasms in the inflamed coronary artery as no infarcts were demonstrated.

To conclude, this case highlights that a detailed and thorough post-mortem examination emphasizing on histopathological findings is necessary to identify the cause of sudden death in an otherwise healthy individual. The possibility of Tuberculous coronary arteritis should be considered while dealing with a case of sudden cardiac death in a young person from an endemic region. We reiterate that though TB is a highly prevalent infection, it can present unexpectedly and be a diagnostic challenge.
